# PD‐L1 expression is associated with the spontaneous regression of patients with methotrexate‐associated lymphoproliferative disorders

**DOI:** 10.1002/cam4.4462

**Published:** 2021-11-29

**Authors:** Yuka Gion, Misato Doi, Yoshito Nishimura, Tomoka Ikeda, Midori Filiz Nishimura, Misa Sakamoto, Yuria Egusa, Asami Nishikori, Azusa Fujita, Noriko Iwaki, Naoya Nakamura, Tadashi Yoshino, Yasuharu Sato

**Affiliations:** ^1^ Division of Pathophysiology Okayama University Graduate School of Health Sciences Okayama Japan; ^2^ Division of Clinical Laboratory Hiroshima Red Cross Hospital & Atomic‐bomb Survivors Hospital Hiroshima Japan; ^3^ Department of General Medicine Okayama University Graduate School of Medicine, Dentistry and Pharmaceutical Sciences Okayama Japan; ^4^ Department of Medicine John A. Burns School of Medicine University of Hawai'i Honolulu Hawaii USA; ^5^ Department of Pathology Okayama University Graduate School of Medicine, Dentistry and Pharmaceutical Sciences Okayama Japan; ^6^ Department of Hematology Faculty of Medicine Institute of Medical, Pharmaceutical and Health Sciences Kanazawa University Kanazawa Japan; ^7^ Department of Pathology Tokai University School of Medicine Kanagawa Japan

**Keywords:** classic Hodgkin lymphoma, diffuse large B‐cell lymphoma, methotrexate‐associated lymphoproliferative disorder, programmed cell death‐ligand 1, rheumatoid arthritis

## Abstract

**Background:**

Most patients with methotrexate‐associated lymphoproliferative disorder (MTX‐LPD) show diffuse large B‐cell lymphoma (DLBCL) or classic Hodgkin lymphoma (CHL) types. Patients with MTX‐LPD often have spontaneous remission after MTX discontinuation, but chemotherapeutic intervention is frequently required in patients with CHL‐type MTX‐LPD. In this study, we examined whether programmed cell death‐ligand 1 (PD‐L1) expression levels were associated with the prognosis of MTX‐LPD after MTX discontinuation.

**Methods:**

A total of 72 Japanese patients diagnosed with MTX‐LPD were clinicopathologically analyzed, and immunohistochemical staining of PD‐L1 was performed in 20 DLBCL‐type and 24 CHL‐type MTX‐LPD cases to compare with the clinical course.

**Results:**

PD‐L1 was expressed in 5.0% (1/20) of patients with DLBCL‐type MTX‐LPD, whereas it was expressed in 66.7% (16/24) of the patients with CHL‐type MTX‐LPD in more than 51% of tumor cells. Most CHL‐type MTX‐LPD patients with high PD‐L1 expression required chemotherapy owing to exacerbations or relapses after MTX discontinuation. However, no significant differences in clinicopathologic findings at diagnosis were observed between PD‐L1 high‐ and low‐expression CHL‐type MTX‐LPD.

**Conclusion:**

PD‐L1 expression was significantly higher in patients with CHL‐type than DLBCL‐type MTX‐LPD, suggesting the need for chemotherapy in addition to MTX discontinuation in CHL‐type MTX‐LPD patients to achieve complete remission. No association was observed between PD‐L1 expression levels and clinical findings at diagnosis, suggesting that PD‐L1 expression in tumor cells influences the pathogenesis of CHL‐type MTX‐LPD after MTX discontinuation.

## Graphical abstract

Patients with classic Hodgkin lymphoma (CHL)‐type methotrexate‐associated lymphoproliferative disorder (MTX‐LPD) with high programmed cell death‐ligand 1 (PD‐L1) expression tended to have exacerbations and relapses after MTX discontinuation. Our study suggests that the PD‐1/PD‐L1 pathway may be involved in refractoriness to MTX discontinuation in CHL‐type MTX‐LPD.

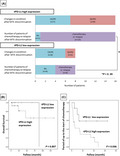

## 1 INTRODUCTION

Methotrexate (MTX) is an immunosuppressive antifolate frequently used as an antirheumatic agent. Although MTX is widely used as an “anchor drug” for rheumatoid arthritis (RA) worldwide, its adverse effects include myelosuppression, interstitial pneumonia (MTX pneumonia), and infectious diseases related to immunosuppression. Furthermore, in recent years, the number of cases reporting MTX‐associated lymphoproliferative disorders (MTX‐LPD) has increased rapidly in Japan.^1^, ^2^, ^3^, ^4^, ^5^, ^6^ MTX‐LPD is also classified as “other iatrogenic immunodeficiency‐associated lymphoproliferative disorder” in the 4th edition of the World Health Organization classification.^7^ However, it is difficult to determine whether MTX‐LPD is caused by the autoimmune disease or the treatment with immunosuppressive drugs. Various risk factors, including chronic infection with Epstein–Barr virus (EBV), older age, and disease activity of RA, have been reported.^8^, ^9^, ^10^, ^11^ A Japanese study reported that treatment with immunosuppressive agents, such as MTX and tacrolimus, can increase the risk of lymphoma development in patients with RA.^8^

MTX‐LPD exhibits various histopathological features. The most commonly reported histological subtype is diffuse large B‐cell lymphoma (DLBCL; 35%–60%), followed by classic Hodgkin lymphoma (CHL; 12%–25%).^7^ Although MTX‐LPD has similar histopathology to that of de novo lymphoma, many patients exhibit spontaneous remission after MTX discontinuation.^4^, ^12^, ^13^ We previously reported for the first time that patients with CHL‐type MTX‐LPD are less likely to exhibit remission after MTX discontinuation and require additional chemotherapy.^14^ We also found that CHL‐type MTX‐LPD more frequently involves extranodal sites as compared to that of de novo CHL. However, the underlying pathophysiology remains unclear.

The programmed cell death‐1 (PD‐1)/programmed cell death‐ligand 1 (PD‐L1) pathway in various tumors have recently attracted attention. PD‐L1 is highly expressed in Hodgkin/Reed‐Sternberg (HRS) cells found in de novo CHL.^15^, ^16^, ^17^ Mutations in the *PD‐L1* and *PD‐L2* loci are characteristic of de novo CHL, and 9p24.1 gene amplification is more prominently detected in advanced‐stage patients. Progression‐free survival is significantly shorter in patients with 9p24.1 amplification.^18^ In addition, a recent study noted that anti‐PD‐1 antibody could be an effective treatment option for de novo CHL.^19^ Therefore, we investigated the relationship between PD‐L1 expression and clinical features of DLBCL‐type and CHL‐type MTX‐LPD. This study focused on examining the role of PD‐1/PD‐L1 in CHL‐type MTX‐LPD prognosis after MTX discontinuation.

## 2 MATERIALS AND METHODS

### 2.1 Patients

**FIGURE 1:**
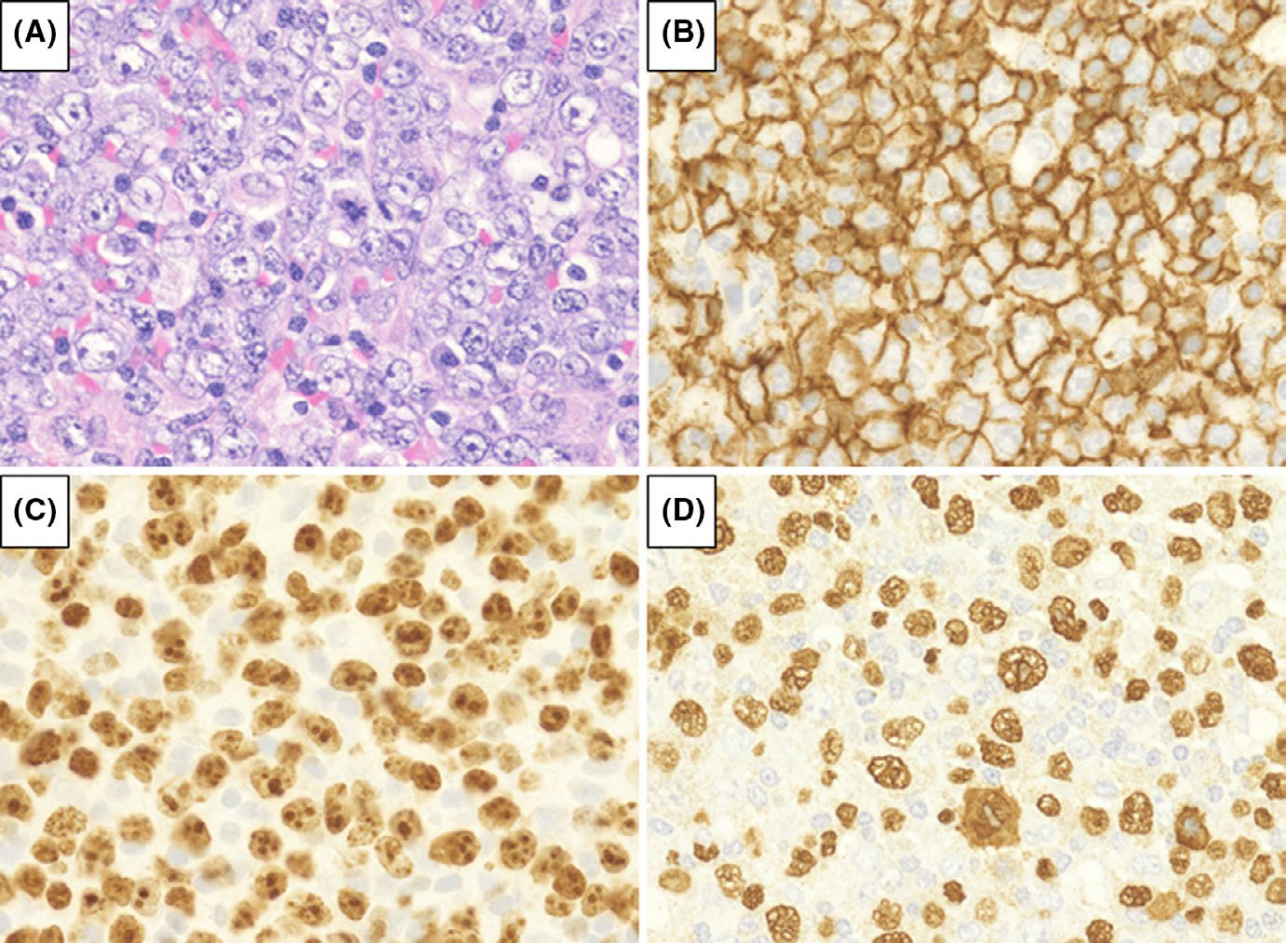
Morphological definition of diffuse large B‐cell lymphoma‐type methotrexate‐associated lymphoproliferative disorders. (A) Hematoxylin and eosin‐stained specimens showed large atypical lymphoid cells exhibiting monomorphic and sheet‐like proliferation. Tumor cells were (B) CD20‐positive, (C) had a high Ki‐67 labeling index and (D) were also positive for Epstein–Barr Virus‐encoded small RNA in situ hybridization

**FIGURE 2:**
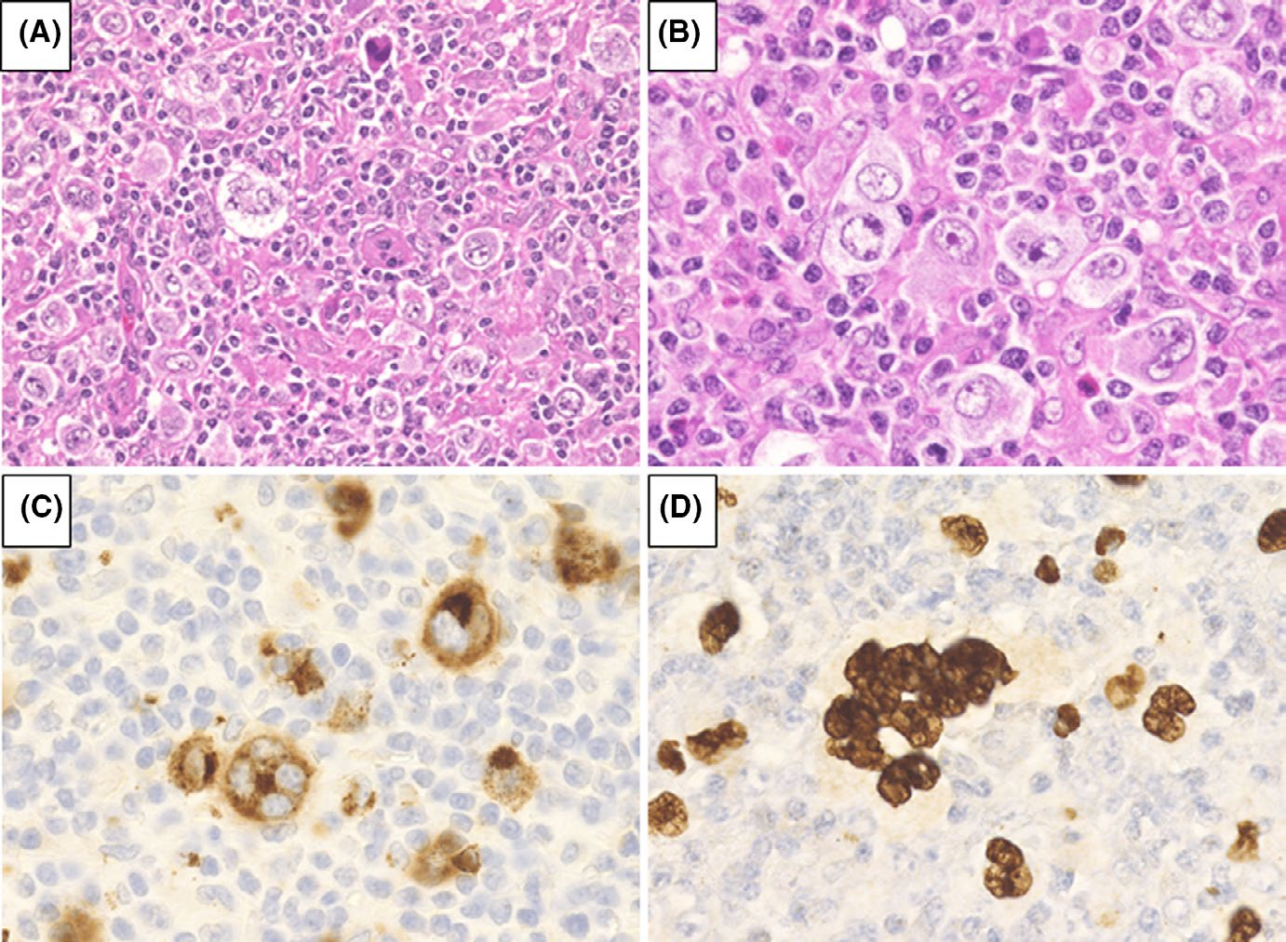
Morphological definition of classic Hodgkin lymphoma‐type methotrexate‐associated lymphoproliferative disorders. (A) Hematoxylin and eosin staining at low‐power field and (B) at high‐power field. Hodgkin and Reed‐Sternberg (HRS) cells were observed in cellular backgrounds rich in lymphocytes and histiocytes. HRS cells were (C) CD30‐positive and (D) positive for Epstein–Barr virus‐encoded small RNA in situ hybridization

We analyzed 72 patients who were diagnosed with MTX‐LPD; 39 patients had DLBCL‐type MTX‐LPD (Figure 1), whereas the remaining 33 had CHL‐type MTX‐LPD (Figure 2). The Eastern Cooperative Oncology Group performance status, clinical stage (CS), International Prognostic Score, and laboratory data were collected from medical records.

The study protocol was approved by the Institutional Review Board of Okayama University (reference numbers: 1607‐016 and 2007‐033). Comprehensive informed consent was obtained from all patients through an opt‐out methodology at Okayama University.

### 2.2 Histological examination

Tissue specimens were fixed in 10% formaldehyde, embedded in paraffin, and cut into 3‐μm thick sections. Each section was further stained with hematoxylin and eosin. Immunohistochemical staining was performed on paraffin sections using antibodies against CD3 (1:200, LN10; Novocastra), CD5 (1:100, 4C7; Novocastra), CD10 (1:100, 56C6; Novocastra), CD15 (1:50, Carb‐3; DAKO), CD20 (1:100, L26; DAKO), CD30 (1:40, Ber‐H2; DAKO), CD79a (1:100, JCB117; DAKO), Ki‐67 (1:2500, MIB‐1; DAKO), and PD‐L1 (1:400, E1LN3; Cell Signaling Technology) and samples were analyzed using a Bond III Stainer (Leica Biosystems).

### 2.3 Evaluation of PD‐L1 expression

**FIGURE 3:**
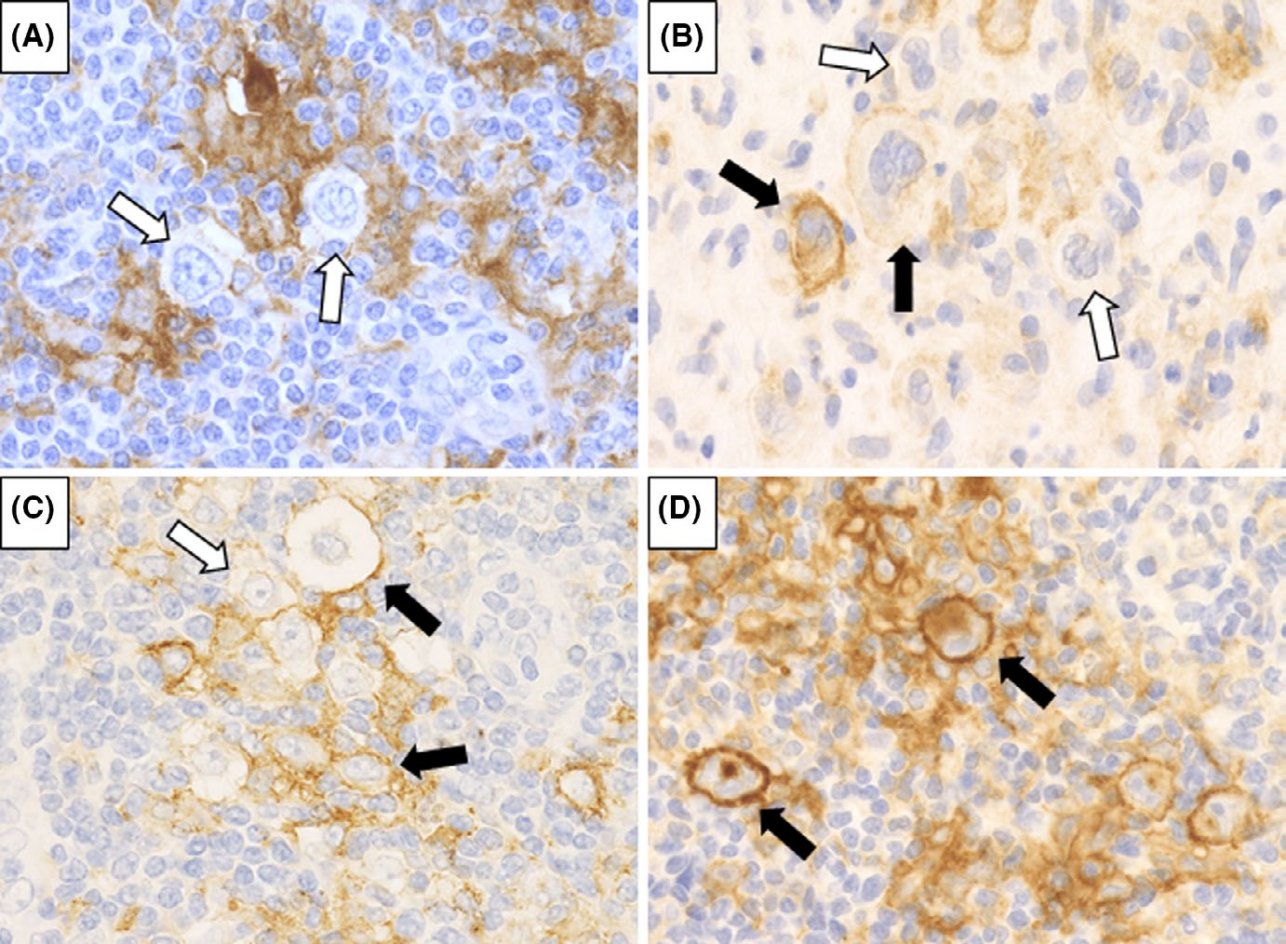
Evaluation of programmed cell death‐ligand 1 (PD‐L1) positive scores in classic Hodgkin lymphoma‐type methotrexate‐associated lymphoproliferative disorders. The evaluation was scored as follows: (A) score 1 for PD‐L1‐positive rate ≤25%, (B) score 2 for 26%–50%, (C) score 3 for 51%–75%, and (D) score 4 for ≥76%. Black arrow indicates PD‐L1‐positive Hodgkin and Reed‐Sternberg (HRS) cells and white arrow indicates PD‐L1‐negative HRS cells

The expression of PD‐L1 was examined in 20 DLBCL‐type and 24 CHL‐type MTX‐LPD cases and the percentage of PD‐L1‐positive tumor cells was recorded in each case. Cases were scored and classified into four categories depending on the percentage of PD‐L1‐positive tumor cells: score 1, PD‐L1‐positive rate ≤25%; score 2, 26%–50%; score 3, 51%–75%; and score 4, ≥76% (Figure 3). Cases were defined as “PD‐L1 high expression group” if the patient had a PD‐L1‐positive rate ≥51% (score 3 or 4) and were defined as “PD‐L1 low expression group” for cases with PD‐L1‐positive rate ≤50% (score 1 or 2).

Cells in the microenvironment, such as histiocytes, also express PD‐L1; therefore, the specimens were also analyzed for PD‐L1 expression in the microenvironment. Cases were defined as “microenvironment PD‐L1 positive” when accompanied by non‐neoplastic PD‐L1‐positive cells. Microenvironment PD‐L1 expression was differentiated from tumor cell expression by evaluating morphology, low nuclear/cytoplasmic ratio, and non‐atypical nuclei without atypia.

### 2.4 Detection of EBV infection pattern in CHL‐type MTX‐LPD

Immunohistochemical staining of latent membrane protein 1 (LMP1; 1:50, CS1‐4, Novocastra), EBV nuclear antigen 2 (EBNA2; 1:100, PE2, Abcam), and in situ hybridization of EBV‐encoded small RNA (EBER1; Novocastra) were performed using an automated Bond III Stainer.

EBV infection patterns were classified into three types based on the results of immunostaining and in situ hybridization: type I (EBER‐positive, LMP1‐negative, EBNA2‐negative); type II (EBER‐positive, LMP1‐positive, EBNA2‐negative); and type III (EBER‐positive, LMP1‐positive, EBNA2‐positive).

### 2.5 Statistical analyses

Differences between laboratory data and patient characteristics at each CS were analyzed using the Mann–Whitney test. The correlation between the PD‐L1‐positive rate and clinical course after MTX discontinuation was determined using Fisher's exact test.

Overall survival was defined as the time from MTX‐LPD diagnosis to death or final follow‐up. The period up to chemotherapy start was defined as the time from MTX‐LPD diagnosis to chemotherapy start or death or final follow‐up. Follow‐up duration was calculated as the time of MTX‐LPD diagnosis to death from any cause or final follow‐up. Survival curves were evaluated using the Kaplan–Meier method and compared using log rank tests. Statistical analyses were performed using SPSS (version 14.0; SPSS).

## 3 RESULTS

### 3.1 Patient characteristics

All patients received MTX; 33 patients received MTX monotherapy, 26 patients received a combination of prednisolone and MTX, and 19 patients received MTX with other disease‐modifying antirheumatic drugs such as infliximab, etanercept, adalimumab, tacrolimus, or bucillamine.

The median age of the entire study was 67 years (32–84 years), and there was no difference between MTX‐LPD subtypes. There was a higher proportion of female patients than male patients in both subtypes (Table 1). The Eastern Cooperative Oncology Group performance status and CS were not significantly different between MTX‐LPD subtypes. A CS ≥3 was observed in 74.4% (29/39) of patients with DLBCL‐type and 72.7% (24/33) of patients with CHL‐type MTX‐LPD (Table 1).

**TABLE 1:** Clinical characteristics

	All patients (*n* = 72)	DLBCL‐type (*n* = 39)	CHL‐type (*n* = 33)	*p*‐value
Age	67 (32–84)	69 (55–82)	64 (32–84)	0.08
Sex (male/female)	21/51	10/29	11/22	0.60
PS ≥2	28.8% (17/59)	38.7% (12/31)	17.9% (5/28)	0.09
Clinical stage ≥3	73.6% (53/72)	74.4% (29/39)	72.7% (24/33)	1.00
Extranodal disease	45.8% (33/72)	56.4% (22/39)	33.3% (11/33)	0.06
Extranodal disease ≥2	12	6	6	
LDH above normal	66.2% (45/68)	74.3% (26/35)	57.6% (19/33)	0.20
sIL−2R above normal	86.2% (56/65)	90.9% (30/33)	81.3% (26/32)	0.30
B symptom positive	53.4% (31/58)	53.1% (17/32)	53.8% (14/26)	1.00
EBER positive	88.1% (59/67)	85.3% (29/34)	90.9% (30/33)	0.71
Treatment for autoimmune disease				
MTX	100%			
Prednisolone	36.1% (26/72)	23.1% (9/39)	51.5% (17/33)	0.02
Other immunosuppressive drugs	26.4% (19/72)	25.6% (10/39)	27.3% (9/33)	1.00
Clinical response to MTX discontinuation				0.09
Complete remission/partial remission	74.2% (49/66)	84.8% (28/33)	63.9% (21/33)	
Stable disease/progressive disease	25.8% (17/66)	15.2% (5/33)	36.4% (12/33)	
Additional treatment after diagnosis of MTX‐LPD				0.004
No therapy	52.8% (38/72)	69.2% (27/39)	33.3% (11/33)	
Chemotherapy	47.2% (34/72)	30.8% (12/39)	66.7% (22/33)	
Clinical response to additional chemotherapy				0.39
Complete response	82.3% (28/34)	91.7% (11/12)	77.2% (17/22)	
Progressive disease	17.6% (6/34)	8.3% (1/12)	22.7% (5/22)	
Outcome				0.72
Alive	63	35	28	
Died	9	4	5	

Note

High lactate dehydrogenase (LDH) levels and soluble interleukin‐2 receptor (sIL‐2R) levers were defined as values equal to or greater than the reference value.

Abbreviations: CHL, classic Hodgkin lymphoma; DLBCL, diffuse large B‐cell lymphoma; EBER, Epstein–Barr Virus‐encoded small RNA in situ hybridization; MTX, methotrexate; PS, Eastern Cooperative Oncology Group performance status.

### 3.2 Clinicopathological characteristics of DLBCL‐type MTX‐LPD

#### 3.2.1 Clinical characteristics of DLBCL‐type MTX‐LPD

The median age of patients with DLBCL‐type MTX‐LPD was 69 years (55–82 years) with a predominance of females (29/39; 74.4%). Twelve of these patients (12/31; 30.8%) had an Eastern Cooperative Oncology Group performance status ≥2. Twenty‐two of the patients (22/39; 56.4%) had extranodal lesions at sites such as the lung, liver, bone marrow, kidney, adrenal gland, and skin. The percentage of patients with CS III or IV was 74.4% (29/39).

Of the 39 patients with DLBCL‐type MTX‐LPD, 38 had RA, and 1 had both polymyositis and Sjögren's syndrome. All patients were taking MTX for these autoimmune diseases, and 18 of the patients were also taking other anti‐RA drugs such as prednisolone, bucillamine, tacrolimus, etanercept, adalimumab, infliximab, salazosulfapyridine, and celecoxib.

The median lactate dehydrogenase (LDH) level at the time of MTX‐LPD diagnosis was 329 IU/L (168–880 IU/L), with 74.3% (26/35) of patients showing higher levels than the upper normal limit. The median soluble interleukin‐2 receptor (sIL‐2R) level was 2604 U/ml (252–40,727 U/ml), with 90.9% (30/33) of patients showing a higher level than the reference value (Table 1).

#### 3.2.2 Clinical courses after MTX discontinuation in DLBCL‐type MTX‐LPD

In 33 out of 39 patients with DLBCL‐type MTX‐LPD, MTX treatment was discontinued after the diagnosis of MTX‐LPD. Of these patients, 27/33 (81.8%) showed spontaneous disappearance of lesions after MTX discontinuation and did not need chemotherapy. Additionally, one patient had partial remission and subsequently received chemotherapy. In 5/33 (15.1%) patients the lesions did not regress or even progressed after MTX discontinuation, and these patients received treatment equivalent to chemotherapy for de novo DLBCL. Of those who received additional chemotherapy, 4/5 (80.0%) experienced remission and 1/5 (20.0%) died owing to secondary leukemia.

Moreover, 6/39 patients with DLBCL‐type MTX‐LPD were critically ill at the time of diagnosis, for which they required immediate chemotherapy in addition to MTX discontinuation. After chemotherapy, 5/6 (83.3%) of these patients exhibited remission, and one died owing to DLBCL‐type MTX‐LPD.

#### 3.2.3 Immunohistochemical phenotype

Atypical lymphoid cells showed monomorphic and diffused proliferation (Figure 1). Immunohistochemical staining showed that 0% (0/39), 7.4% (2/27), 3.3% (3/24), and 89.7% (35/39) of samples were positive for CD3, CD5, CD10, and CD20, respectively. All cases of DLBCL‐type MTX‐LPD showed a high Ki‐67 labeling index (>30%), with an EBER‐positive rate of 85.3% (Table 1).

### 3.3 Clinicopathological characteristics of CHL‐type MTX‐LPD

#### 3.3.1 Clinical characteristics of CHL‐type MTX‐LPD

The clinical data of 33 patients with CHL‐type MTX‐LPD analyzed in this study are shown in Tables 1 and 2. The median age was 64 years (32–84 years), with a predominance of females (22/33; 66.7%).

**TABLE 2:** Clinical findings of patients with classic Hodgkin lymphoma‐type methotrexate‐associated lymphoproliferative disorders

Case no. Age/sex	Primary immune disorders	Immunomodulator	Extranodal involvement site	Clinical stage	EBV latency pattern	Response after MTX discontinuation	Relapse after MTX discontinuation	Chemo therapy	Outcome, follow‐up duration
1 45/F	RA, SS	MTX, PSL	Bone mallow	Ⅳ	Ⅱ	PD		Rituximab, ADR, CY	PD, 3 months (dead)
2 63/M	RA	MTX, etanercept		Ⅲ	Ⅱ	SD		ABVd	CR, 11.3 years
3 69/F	RA	MTX	Cerebellum	Ⅰ	Ⅱ	PD		ABVd	CR, 7.4 years (dead)
4 79/F	RA	MTX		Ⅲ	Ⅱ	PR	+	ABVD	CR, 1.8 years
5 68/F	RA	MTX, PSL, tacrolimus hydrate		Ⅱ	U.D.	CR	+	ABVD	CR, 9.5 years
6 65/F	RA	MTX, PSL		Ⅲ	Ⅱ	SD		ABVd	CR, 9.4 months
7 63/M	RA	MTX, PSL, bucillamine		Ⅰ	Ⅱ	SD		ABVd	CR, 3 months
8 63/F	RA	MTX	Thoracic spine, liver	Ⅳ	U.D.	PR	+	ABVD	Relapse after chemotherapy, 20 years (dead)
9 84/M	RA	MTX, PSL, tacrolimus hydrate		Ⅲ	none	SD		ABVD	CR, 9.8 months
10 59/F	RA	MTX		Ⅰ	Ⅱ	PR	+	ABVD	CR, 5.5 years
11 53/F	RA	MTX, bucillamine, gold sodium thiomalate		Ⅲ	Ⅱ	PR	+	ABVd	U.D., 3.8 years (dead)
12 64/F	RA	MTX, adalimumab, PSL	Brain, lung	Ⅳ	U.D.	CR			CR, 1.7 years
13 63/F	RA	MTX, infliximab		Ⅲ	U.D.	PR	+	ABVd	CR, 1.6 years
14 32/F	RA, SS	MTX, PSL	liver	Ⅳ	Ⅱ	SD		ABVD	CR, 5.2 years
15 63/F	RA	MTX	Bone mallow, liver	Ⅳ	Ⅱ	PR	+	ABVd	PD, 2.1 years
16 83/M	RA	MTX, PSL		Ⅰ	Ⅱ	CR			CR, 5.1 years
17 84/F	RA	MTX		Ⅲ	Ⅱ	CR			CR, 1 year
18 81/M	RA	MTX		Ⅲ	U.D.	PR			PR, 6.3 months
10 63/M	Psoriatic arthritis	MTX	skin	Ⅳ	Ⅱ	PR			PR, 7.1 months
20 68/F	RA	MTX, PSL	Pharynx, adrenal glands	Ⅳ	Ⅱ	PR			PR, 2.1 months
21 56/F	RA	MTX	liver	Ⅳ	none	PR	+	ABVD	CR, 7.6 months
22 70/M	RA	MTX, iguratimod, bucillamine	Bone mallow, lung	Ⅳ	Ⅱ	PR			PR, 2.7 years
23 57/M	RA	MTX, PSL		Ⅱ	Ⅱ	PD		ABVD	PD, 1 year
24 63/F	RA, chronic thyroiditis	MTX, PSL		Ⅲ	Ⅱ	PR	+	ABVd	CR, 2.1 years
25 71/F	RA	MTX, PSL		Ⅲ	Ⅱ	PR	+		PR, 1.7 years
26 67/F	RA, scleroderma	MTX		Ⅱ	Ⅰ	SD		ABVD	PD, 1.9 years (dead)
27 64/F	RA	MTX	Lung, spleen, nasopharynx	Ⅳ	Ⅱ	PR			CR 1.9 years
28 73/M	RA	MTX, PSL		Ⅲ	Ⅱ	PD		A‐AVD	Following, 1.7 months
29 71/F	RA	MTX, PSL		Ⅲ	Ⅱ	PR	+	ABVd	CR, 1 year
30 50/M	RA	MTX, PSL		Ⅲ	Ⅱ	PD			PD, 2.4 months
31 48/F	RA	MTX, tacrolimus hydrate, PSL		Ⅲ	none	SD		ABVd	CR, 9 years
32 41/M	SAPHO syndrome	MTX, PSL		Ⅰ	Ⅱ	CR	+	ABVD	CR, 6 years
33 73/F	RA	MTX		Ⅰ	U.D.	PR			CR, 1 month

Abbreviations: ABVD, Adriamycin + bleomycin + vinblastine + dacarbazine; ADR, doxorubicine; CR, complete response; CY, cyclophosphamide; MTX, methotrexate; PD, progressive disease; PR, partial response; PSL, prednisolone; RA, rheumatoid arthritis; SD, sable disease; SS, Sjögren's syndrome.

Of the 33 patients with CHL‐type MTX‐LPD, 31 had RA and of these 31 patients, two had Sjögren's syndrome, one had scleroderma, and one had chronic thyroiditis. The other two patients without RA had either psoriatic arthritis or SAPHO syndrome.

All patients were taking MTX for these autoimmune diseases, and 21/33 (63.6%) patients were also taking other anti‐RA drugs such as prednisolone, bucillamine, tacrolimus, iguratimod, etanercept, adalimumab, or infliximab.

The patients underwent biopsies of either the lymph nodes, bone marrow, cerebellum, skin, or nasopharynx. Extranodal lesions were observed in 11/33 (33.3%) patients, including in the lung, liver, bone marrow, pharynx, kidney, adrenal gland, cerebellum, and skin, and two or more extranodal lesions were observed in 6/11 (54.5%) patients. Of the 33 patients, 24/33 (72.8%) were CS III or IV and 5/28 (17.9%) had an Eastern Cooperative Oncology Group performance status of ≥2.

Anemia with hemoglobin levels <10.5 g/dl was observed in 9/30 (30%) patients with CHL‐type MTX‐LPD. Low total lymphocyte count (<600/mm^2^ or 8% of leukocytes) was observed in 17.6% (5/28) of the patients. The median LDH level at the time of MTX‐LPD diagnosis was 230 IU/L (114–465 IU/L), with 57.6% (19/33) of the patients showing higher levels than the upper normal limit. In addition, sIL‐2R levels were measured in 32 cases. The median value of sIL‐2R was 1806 U/ml (233–18,900 U/ml), and 26/32 (81.3%) patients showed higher values than the reference values (Table 1).

#### 3.3.2 Clinical courses after MTX discontinuation in CHL‐type MTX‐LPD

In all patients with CHL‐type MTX‐LPD, MTX was discontinued immediately after the diagnosis of MTX‐LPD. Spontaneous remission was observed in 9/33 (27.3%) patients after MTX discontinuation, and these patients had no relapse; therefore, they did not require chemotherapy (Table 2).

Relapse after lesion reduction was observed in 12/33 patients (36.4%) and 11 (91.7%) of these relapsed patients received similar chemotherapy to that for de novo CHL (such as ABVD and ABVd). Subsequently, 9/11 (81.8%) of these patients experienced complete remission, and the other two (18.2%) exhibited disease progression or died. One patient did not receive chemotherapy given her age and immunocompromised status owing to long‐term RA treatment.

Despite MTX discontinuation, 12/33 (36.4%) patients with CHL‐type MTX‐LPD showed disease progression. Of these patients, 7/12 (58.3%) experienced complete remission after chemotherapy, two patients died during chemotherapy, one patient did not respond to chemotherapy, one patient was being followed after chemotherapy, and one patient was being followed without undergoing chemotherapy.

A total of 22/33 (66.7%) patients with CHL‐type MTX‐LPD analyzed in this study received chemotherapy after MTX discontinuation, and the median length of time up to the start of chemotherapy was 2.5 months. In addition, 5/33 (15.1%) patients died during or after chemotherapy. The cause of death included cholecystitis, bacterial pneumonia, and disseminated intravascular coagulation syndrome.

No significant differences in LDH and sIL‐2R levels were observed between the stage I/II and III/IV groups. In addition, we also examined the correlation between LDH and sIL‐2R values and that of the clinical response after MTX discontinuation; no statistically significant differences were observed.

#### 3.3.3 Immunohistochemical phenotype

In all cases of patients with CHL‐type MTX‐LPD, the histopathological morphology was similar to that of de novo CHL. HRS cells were observed and found to be CD30‐positive and partially CD15‐positive or ‐negative (Figure 2). In addition, 13/32 (40.6%) patients were CD20‐positive, and 10/27 (37.0%) patients were CD79a‐positive.

It was difficult to distinguish between polymorphic B‐cell LPD and CHL‐type because CD30‐positive HRS cell was frequently observed in polymorphic B‐cell LPD. However, polymorphic B‐cell LPD was frequently accompanied by CD20‐positive medium to large sized atypical monomorphic cells, which were not observed in CHL‐type. Based on these findings, we diagnosed CHL‐type in this study.

The nuclei of HRS cells were positive for EBER (Figure 2D) and the positive rates for EBER, LMP1, and EBNA2 were 93.5% (29/31), 82.6% (24/29), and 0% (0/27), respectively. EBV latency pattern was analyzed in 25 cases; 2 cases had type I, 23 cases had type II, and none had a type III pattern (Table 2).

### 3.4 Analysis of PD‐L1 expression

#### 3.4.1 Relationship between PD‐L1 expression and clinical course in DLBCL‐type MTX‐LPD

**FIGURE 4:**
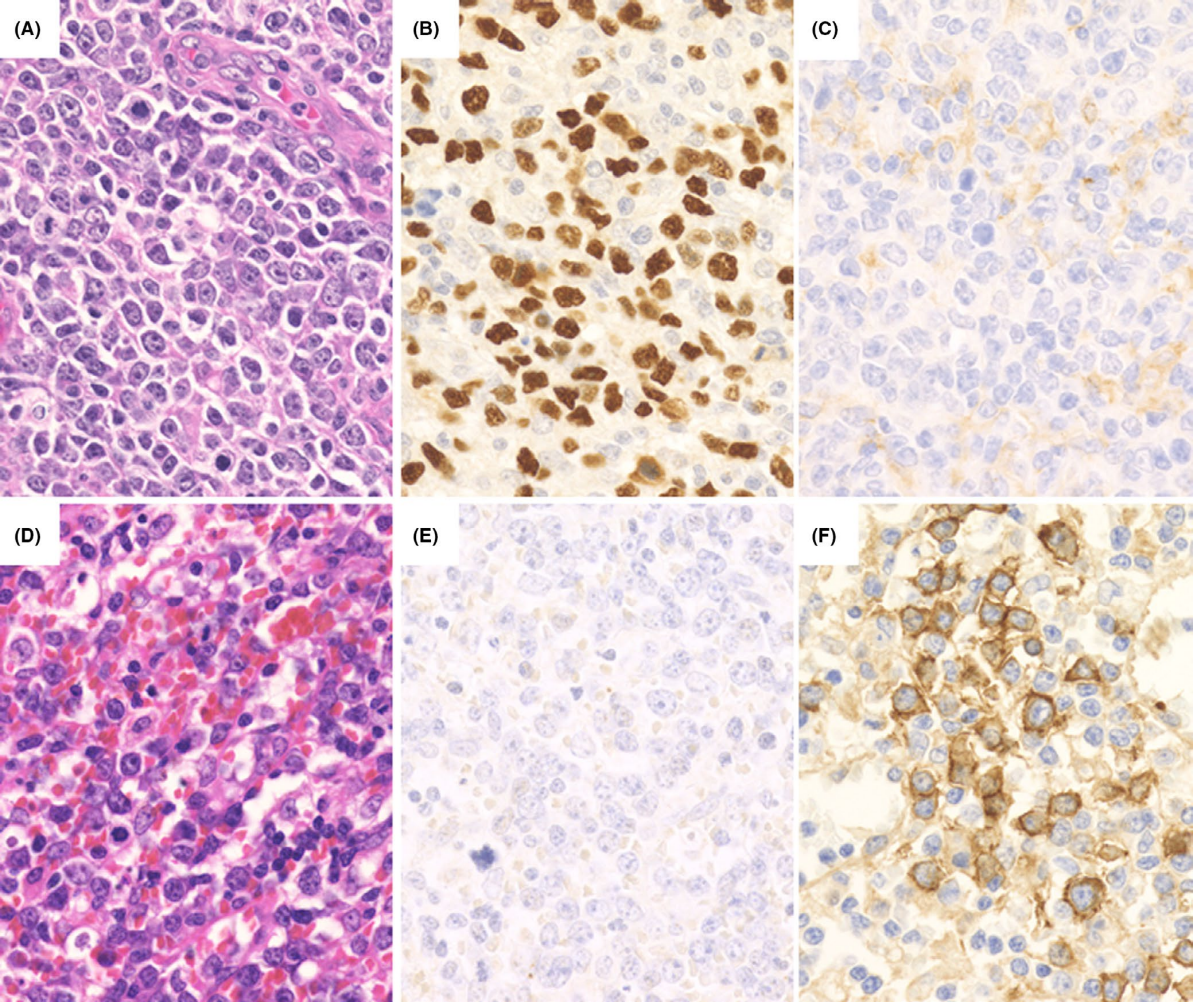
Programmed cell death‐ligand 1 (PD‐L1) staining results in diffuse large B‐cell lymphoma‐type methotrexate‐associated lymphoproliferative disorders (DLBCL‐type MTX‐LPD). DLBCL‐type MTX‐LPD with PD‐L1 score 1: (A) hematoxylin and eosin staining showing tumor cells (B) positive for Epstein–Barr virus‐encoded small RNA (EBER) in situ hybridization and (C) weakly positive PD‐L1 cells (score 1). DLBCL‐type MTX‐LPD with PD‐L1 score 4: (D) hematoxylin and eosin staining showing tumor cells (E) negative for EBER, (F) partially strongly positive, and diffusely weakly positive for PD‐L1

Of the 20 patients with DLBCL‐type MTX‐LPD, 19 patients had a PD‐L1 positivity rate score of 1 (Table 3; Figure 4A–C). Most tumor cells were PD‐L1‐negative, and a few were weakly PD‐L1‐positive on the membrane; 1/20 patients had a score of 4 (Figure 4D–F). In this patient, tumor cells had a diffuse, weakly PD‐L1‐positive pattern with a scattered strongly PD‐L1‐positive pattern. The prognosis could not be compared owing to the biased expression score of PD‐L1, but one patient with a score of 4 was in a state requiring chemotherapy immediately at the time of MTX‐LPD diagnosis. Although this patient did receive chemotherapy, the patient later died owing to DLBCL‐type MTX‐LPD.

**TABLE 3:** PD‐L1 expression

	DLBCL‐type (*n* = 20)	CHL‐type (*n* = 24)	*p*‐value
Tumor cells			<0.01
Score 1	19	7	
Score 2	0	1	
Score 3	0	6	
Score 4	1	10	
Microenvironment	14	24	0.014

Abbreviations: CHL, classic Hodgkin lymphoma; DLBCL, diffuse large B‐cell lymphoma; PD‐L1, programmed cell death‐ligand 1.

**FIGURE 5:**
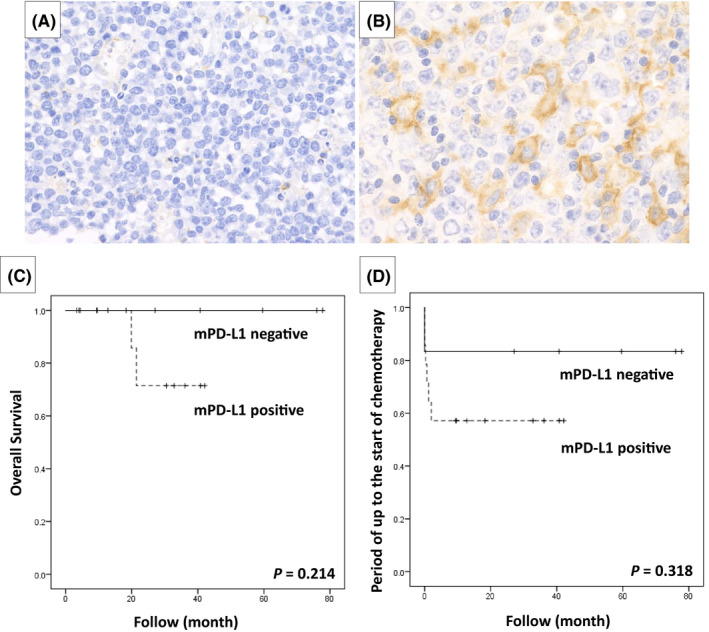
Microenvironment programmed cell death‐ligand 1 (PD‐L1) staining results in diffuse large B‐cell lymphoma‐type methotrexate‐associated lymphoproliferative disorders. (A) Negative and (B) positive cases of microenvironment PD‐L1 (mPD‐L1) expression. The mPD‐L1‐positive cells were mainly histiocytes and fibroblasts. (C) Overall survival and (D) period up to the start of chemotherapy. There was no significant difference between the mPD‐L1‐positive and negative groups

Among DLBCL‐type MTX‐LPD cases, 14/20 (70.0%) had a PD‐L1‐positive microenvironment. Morphologically, it was speculated that histiocytes and fibroblasts could show PD‐L1 positivity in the microenvironment (Table 3; Figure 5A,B). The period up to the start of chemotherapy and overall survival period were examined between the microenvironment PD‐L1‐positive group and the negative group, but no significant difference was observed (Figure 5C,D).

#### 3.4.2 Relationship between PD‐L1 expression and clinical course in CHL‐type MTX‐LPD

**FIGURE 6:**
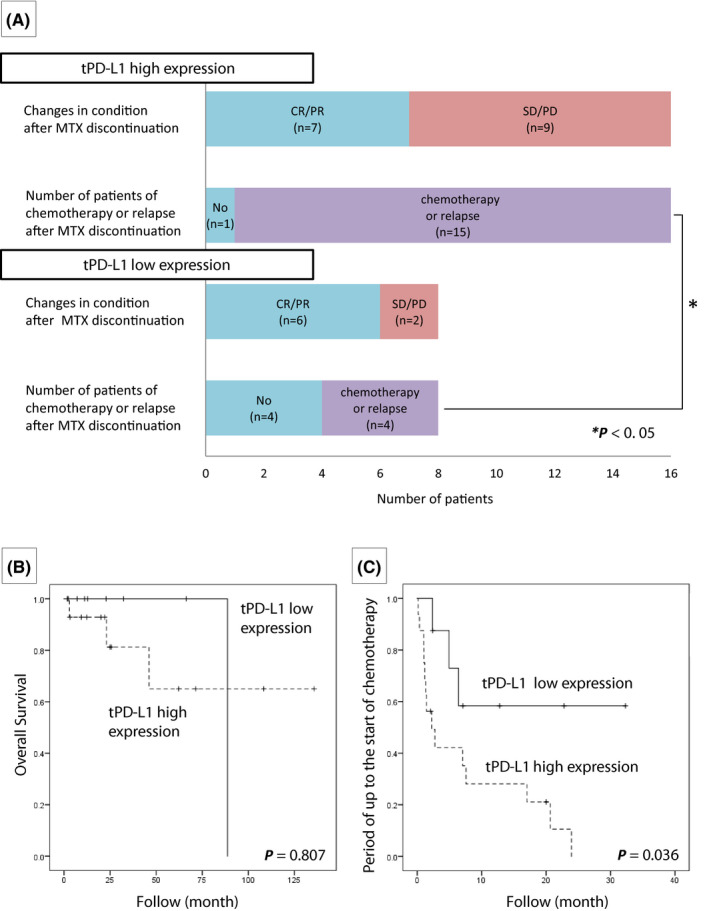
Changes in condition after discontinuation of methotrexate (MTX) in classic Hodgkin lymphoma‐type methotrexate‐associated lymphoproliferative disorders. (A) Lesion changes after MTX discontinuation in tumor cells with high and low programmed cell death‐ligand 1 (PD‐L1; tPD‐L1) expression. There was no significant difference in the effect of lesion reduction between PD‐L1 high‐ and low‐expression groups. However, significantly more cases in the high‐expression group had relapse or needed chemotherapy after MTX discontinuation (*p < 0.05). (B) There was no significant difference in overall survival. (C) In the tPD‐L1 high‐expression group, the period up to the start of chemotherapy was shorter (*p* = 0.036)

In CHL‐type MTX‐LPD cases, all patients with HRS cells were found to be positive for PD‐L1. No case with a PD‐L1‐positive rate of <5% was found. However, even in HRS cells, the expression varied from strong to weak expression. Of the 24 patients with CHL‐type MTX‐LPD examined in this study, 10 patients (41.7%) had a PD‐L1 positivity rate of score 4, 6 (25.0%) had a score of 3, 1 (4.2%) had a score of 2, and 7 (29.1%) had a score of 1 (Table 3). Figure 6A shows the clinical management and prognosis after MTX discontinuation of patients with CHL‐type MTX‐LPD based on PD‐L1 positivity. In the PD‐L1 high‐expression group, patients were less likely to achieve remission, although there was no significant difference compared with that in the PD‐L1 low‐expression group (*p* = 0.210). We also investigated whether relapse or the need for chemotherapy after MTX discontinuation was associated with PD‐L1 expression levels. Our findings showed that there were significantly more relapses in the PD‐L1 high‐expression group than in the PD‐L1 low‐expression group after MTX discontinuation, requiring additional chemotherapy (Figure 6A, *p* < 0.05). However, the overall survival was not significantly different between the PD‐L1 high‐ and low‐expression groups (Figure 6B, *p* = 0.807). Furthermore, the time period until patients received additional chemotherapy was 1.84 months in the PD‐L1 high‐expression group and 3.89 months in the PD‐L1 low‐expression group after MTX discontinuation. The higher the PD‐L1‐positive rate, the shorter the period required for chemotherapy initiation (Figure 6C, *p* = 0.036).

We compared clinical findings, such as performance status and CS at the time of diagnosis of CHL‐type MTX‐LPD, between the groups, that is, the PD‐L1 high‐ and low‐expression groups. However, although there were no characteristic differences in clinical findings between these groups, the sIL‐2R values at the time of diagnosis tended to be higher in the PD‐L1 high‐expression group than in the PD‐L1 low‐ expression group (Table 4).

**TABLE 4:** Clinical data of PD‐L1 high‐expression group and low‐expression group of classic Hodgkin lymphoma‐type methotrexate‐associated lymphoproliferative disorders

	PD‐L1 high expression (*n* = 16)	PD‐L1 low expression (*n* = 8)	*p*‐value
B symptoms	58.3% (7/12)	20% (1/5)	0.294
PS ≥2	18.8% (3/16)	12.5% (1/8)	1.000
CS ≥3	75% (12/16)	75% (6/8)	1.000
Extranodal disease	25% (4/16)	50% (4/8)	0.363
Extranodal disease ≥2	2	2	
Alb (<4.0 g/dl)	66.7% (8/12)	50% (3/6)	0.627
Hb (<10.5 g/dl)	37.5% (6/16)	12.5% (1/8)	0.352
Lymphocyte depletion (<600/mm^3^ or <8% of leukocyte fraction)	21.4% (3/14)	0% (0/7)	0.521
Increased white blood cell count (>15,000/mm^3^)	0% (0/13)	12.5% (1/8)	0.381
LDH			
Median (IU/L)	237 (114–465)	231 (193–286)	
LDH above normal	50.0% (8/16)	12.5% (1/8)	0.178
sIL‐2R			
Median (U/ml)	1938	553.5	
sIL‐2R above normal	86.7% (13/15)	62.5% (5/8)	0.208

Abbreviations: CS, clinical stage; LDH, lactate dehydrogenase; PD‐L1, programmed cell death‐ligand 1; PS, Eastern Cooperative Oncology Group performance status; siL‐2R, soluble interleukin‐2 receptor.

**FIGURE 7:**
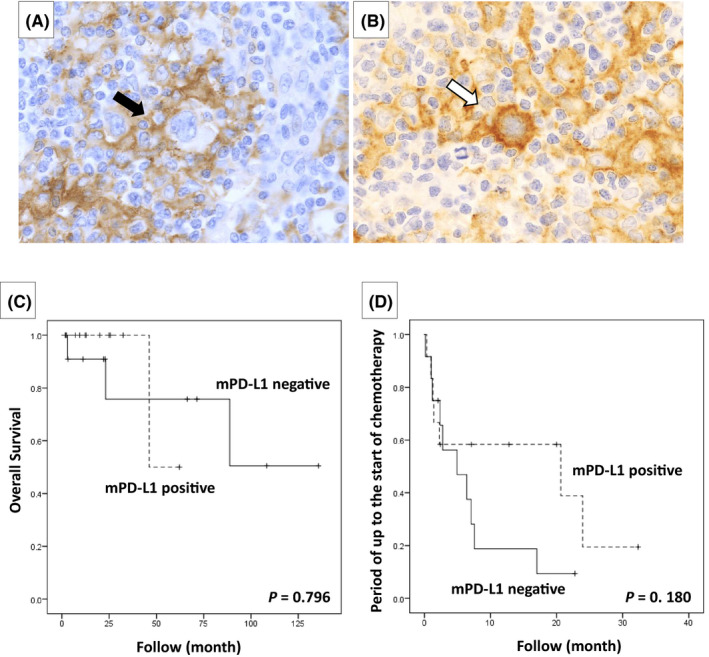
Microenvironment programmed cell death‐ligand 1 (PD‐L1) staining results in classic Hodgkin lymphoma‐type methotrexate‐associated lymphoproliferative disorders. (A) Cases with PD‐L1‐positive histiocytes surrounding Hodgkin and Reed‐Sternberg (HRS) cells (mPD‐L1 positive). However, HRS cells were PD‐L1 negative (black arrow). (B) A case in which a histiocyte around the HRS cells was PD‐L1 negative (mPD‐L1 negative) and the HRS cells strongly expressed PD‐L1 (white arrow). (C) Overall survival and (D) the period of up to the start of chemotherapy were examined between the two groups, but there was no significant difference

In all 24 cases of CHL‐type MTX‐LPD examined, PD‐L1 positivity was also noted in the microenvironment (Table 3). PD‐L1‐positive cells in the microenvironment were mainly macrophages, epithelioid cells, and histiocytes. Furthermore, 12 cases that had PD‐L1‐positive histiocytes with a rosette structure surrounding the HRS cells (Figure 7A) were compared with 12 cases in which histiocytes surrounding HRS cells were PD‐L1‐negative (Figure 7B); no significant difference was observed in the lesion reduction effect after MTX discontinuation between the groups (*p* = 0.457). The overall survival rate and the period until the start of chemotherapy were also examined between the PD‐L1‐positive and ‐negative groups around the HRS cells, but no significant differences were observed (Figure 7C,D).

## 4 DISCUSSION

MTX‐LPD has various tissue morphologies, but the details of each are not fully understood. We previously reported that CHL‐type MTX‐LPD has a poor clinical response after MTX discontinuation and requires an earlier chemotherapeutic intervention than that of DLBCL‐type MTX‐LPD.^14^ Because of the rarity of MTX‐LPD, previous reports are mainly case reports or series. In this study, we analyzed 72 cases diagnosed with MTX‐LPD and examined their clinicopathological features.

Although DLBCL‐type MTX‐LPD showed the same tissue morphology as de novo DLBCL, most cases achieved remission after MTX discontinuation. These patients were less likely to relapse and, hence, did not require chemotherapy. Of the DLBCL‐type MTX‐LPD analyzed in this study, only one case had high‐PD‐L1 expression levels in tumor cells. The patient was seriously ill at the time of MTX‐LPD diagnosis, and chemotherapy was immediately administered; therefore, the effect of MTX discontinuation could not be determined. The patient received a similar chemotherapy regimen as that for de novo DLBCL; nonetheless, the patient died of DLBCL‐type MTX‐LPD. These results indicate that PD‐L1 expression in tumor cells is associated with the clinical outcome of patients after MTX discontinuation. However, the patient was also negative for EBER, which is also associated with poor prognosis^11^, ^20^, ^21^; further investigation is warranted regarding its pathophysiology.

We also analyzed PD‐L1 expression in the tumor microenvironment. The expression rate was similar to that reported by Kohno et al.^22^ However, no association was found between PD‐L1 expression in the microenvironment and the prognosis of patients. According to Kiyasu et al., PD‐L1 expression in tumor cells is correlated with poor prognosis in de novo DLBCL, but PD‐L1 expression in the microenvironment is not.^23^ Based on these results, we suggest that PD‐L1 expression in the microenvironment might not be associated with the spontaneous remission or exacerbation factors in DLBCL‐type MTX‐LPD after MTX discontinuation.

The PD‐L1‐positive rate in HRS cells of de novo CHL is more than 80%, and anti‐PD‐1 antibody therapy for the treatment of de novo CHL is now being adopted.^15^, ^16^, ^17^, ^24^ In this study, we investigated the PD‐L1‐positive rate of HRS cells in CHL‐type MTX‐LPD and found it to be lower than that in de novo CHL. One of the reasons for poor remission of CHL‐type MTX‐LPD after MTX discontinuation can be explained by the possible escape of HRS cells from monitoring by immune cells through the expression of PD‐L1. Furthermore, in this study, we categorized the cases based on PD‐L1 expression (high‐ vs. low‐expression group) and compared PD‐L1 expression intensity and the clinical course of treatment after MTX discontinuation. No significant correlation was found between PD‐L1 expression intensity and lesion disappearance after MTX discontinuation. However, in the PD‐L1 high‐expression group, there were significantly more cases of relapse after discontinuation of MTX and cases requiring chemotherapy as compared with the PD‐L1 low‐expression group. These findings suggest that the higher PD‐L1‐positive rate in HRS cells most likely results in lower remission and requires additional chemotherapy after MTX discontinuation. Additionally, the time required before chemotherapeutic intervention in the PD‐L1 high‐expression group after MTX discontinuation was much shorter (1.84 months) than that in the PD‐L1 low‐expression group (3.89 months) (*p* = 0.036). There was no significant difference in overall survival rate depending on the PD‐L1 expression intensity. Furthermore, clinical findings at the time of diagnosis of CHL‐type MTX‐LPD was compared between the PD‐L1 high‐ and low‐expression groups, but no significant findings were found. For this reason, it is difficult to predict the prognosis from tissue lesions and clinical findings at the time of CHL‐type MTX‐LPD diagnosis. However, the results of this study suggest that PD‐L1 levels affect the pathogenesis of CHL‐type MTX‐LPD. In particular, patients with high expression of PD‐L1 were more likely to require chemotherapy after MTX discontinuation. From the above results, it is likely that PD‐L1 expression in tumors has a strong influence on the clinical course in CHL‐type MTX‐LPD; thus, careful follow‐up is required in cases showing high expression of PD‐L1 at the time of pathological diagnosis.

Furthermore, Kohno et al. reported that immunostaining of PD‐L1 (clone: SP142) was explicitly detected in CHL‐type in MTX‐LPD, whereas tumor cells were PD‐L1‐negative in other histological types.^22^ In addition, they detected an increase in *CD274/PD‐L1* gene copy number in CHL‐type MTX‐LPD. In this study, we investigated PD‐L1 expression using immunohistochemical staining with clone E1LN3. It has been suggested that there are several types of clones in immunohistochemical staining for PD‐L1, which may affect the staining results. Moreover, PD‐L1 expression heterogeneity in tumor tissues also affects the evaluation of PD‐L1 expression. It is known that PD‐L1 expression in tumor cells can change depending on the surrounding environment. When tumor‐specific T‐cells are activated, they produce INF‐γ and attack cancer cells, but cancer cells express PD‐L1 in response to IFN‐γ.^25^ Furthermore, PD‐L1 expression is enhanced by TNF‐α and IL‐1β, suggesting that the expression of PD‐L1 in tumor cells changes depending on the tumor microenvironment. In addition, PD‐L1 expression in the tumor cells of patients may change over time, depending on the disease condition and treatment.

As a genomic abnormality of *PD‐L1*, it is known that the copy number of the 9p24.1 region containing *PD‐L1* and *PD‐L2* is amplified in gastric cancer and malignant lymphoma.^26^ In particular, in de novo CHL, abnormalities in the same area are observed in almost all cases.^18^ In this study, fluorescence in situ hybridization of *PD‐L1/PD‐L2* could not be performed, but it is highly possible that similar copy count abnormalities can be observed in CHL‐type MTX‐LPD. A pathway activated due to a 3ʹ‐untranslated region (UTR) abnormality of the *PD‐L1* gene has been reported by Kataoka et al. as the cause of PD‐L1 overexpression.^27^ The *PD‐L1* gene abnormality is on the C‐terminal side, and in the case of 3ʹ‐UTR abnormality, using an antibody that recognizes the C‐terminal (e.g., clone SP142 and E1LN3) may result in a false negative. Even in the case of companion diagnostics for immune checkpoint inhibitors, as there are various clones and cutoff value settings used for immunostaining, detailed examination is required.

PD‐L1 is expressed in immune cells, such as T‐cells, B‐cells, macrophages, and dendritic cells, in addition to tumor cells. In our study, we also investigated PD‐L1 expression in immune cells within this microenvironment, and examined its role as a predictor of MTX‐LPD prognosis, but we did not find any significant differences. Although not included in this cohort, in the past, in patients who underwent lymph node biopsy after MTX discontinuation at our institution, necrosis in the center of the lesion with surrounding abundant CD8‐positive T‐cells was noted (Figure S1). In Tokuhira et al., the absolute lymphocyte count in the peripheral blood of MTX‐LPD patients recovered rapidly in the spontaneous remission group, but did not change in the exacerbation group.^28^ These recovered lymphocytes were mainly composed of Th1, effector memory B‐cells, and EBV‐specific T‐cells.^2^, ^28^ Based on this analysis and previous reports, we suggest that CD8‐positive T‐cells are involved in the spontaneous regression of MTX‐LPD, and PD‐1/PD‐L1 is a vital molecule for this reaction. Abnormalities in the PD‐1/PD‐L1 pathway may contribute to the poor prognosis for CHL‐type MTX‐LPD caused by excessive PD‐L1 gene amplification.

## 5 CONCLUSIONS

Compared with DLBCL‐type MTX‐LPD, CHL‐type MTX‐LPD was not resolved after MTX discontinuation alone, and required chemotherapy in many cases. In addition, PD‐L1 expression was found to be significantly higher in patients with CHL‐type than in those with DLBCL‐type MTX‐LPD. Patients with CHL‐type MTX‐LPD with high PD‐L1 expression tended to have exacerbations and relapses after MTX discontinuation. Our study suggests that the PD‐1/PD‐L1 pathway is involved in refractoriness to MTX discontinuation in CHL‐type MTX‐LPD.

## CONFLICT OF INTEREST

The authors have no competing interest to declare.

## AUTHOR CONTRIBUTIONS

Conceptualization, Y.G. and Y.S.; methodology, Y.G.; formal analysis, Y.G. and M.D.; investigation, Y.G., M.D., T.I., M.F.N., M.S., Y.E., A.N., A.F., and N.I.; data curation, Y.G.; writing—original draft preparation, Y.G.; writing—review and editing, Y.N., N.N., and Y.S.; supervision, Y.S. and T.Y. All authors have read and agreed to the published version of the manuscript.

## ETHICS STATEMENT

The study protocol was approved by the Institutional Review Board of Okayama University (reference numbers: 1607‐016 and 2007‐033). Comprehensive informed consent was obtained from all patients through an opt‐out methodology at Okayama University.

## Supporting information

Figure S1

## Data Availability

The data might be made available upon request, and some restrictions will apply.
