# Supplementary material for: PD‐L1 expression is associated with the spontaneous regression of patients with methotrexate‐associated lymphoproliferative disorders
Source: Cancer Med. 2021 Nov 29;11(2):417–32. doi: 10.1002/cam4.4462 (PMC8729050; doi:10.1002/cam4.4462)

Supplemental Figure

Histological findings of a patient who underwent lymph node biopsy after MTX discontinuation.

(A) The central part of the tumor was necrotic (on the right) and surrounded by many lymphocytes. (B) Immunohistochemical staining with an anti-CD8 antibody revealed that the surrounding lymphocytes were CD8-positive cytotoxic T-cells.

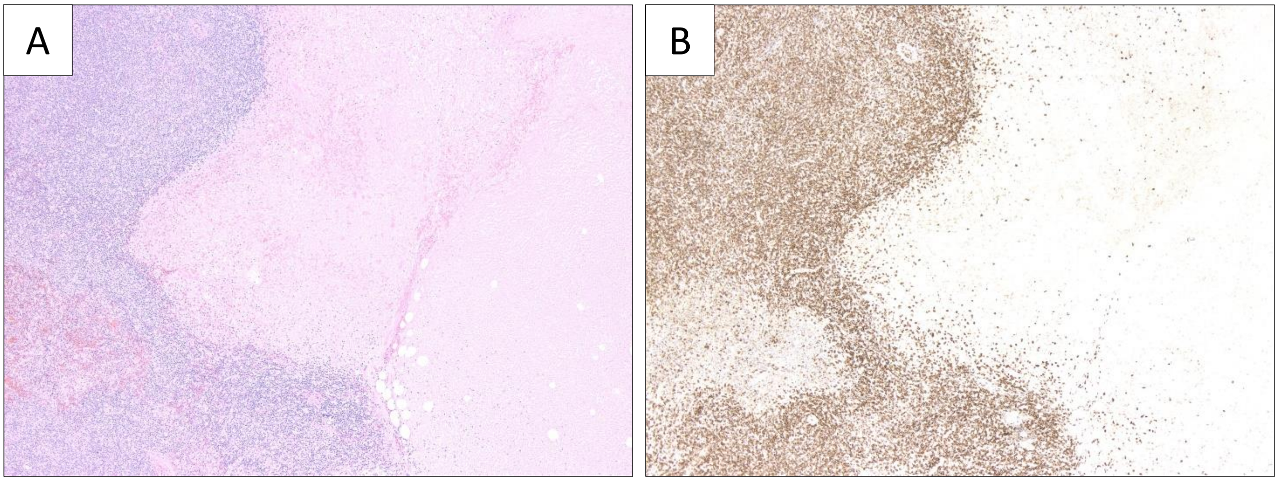

Supplement: Supplementary file 1 — Figure S1 [file CAM4-11-417-s001.pdf]
